# GICPIdb: an archival repository of multimodal data focusing on pathological images for gastrointestinal cancers

**DOI:** 10.3389/fdata.2026.1924721

**Published:** 2026-08-31

**Authors:** Huang Chen, Ling Tong, Jinyang Liu, Kai Liu, Xintao Li, Shufang Shi, Shuxue Xi, Geng Tian, Meijun Zhang, Dingrong Zhong, Shijun Li, Jialiang Yang

**Affiliations:** 1 Department of Pathology, China-Japan Friendship Hospital, Beijing, China; 2 Department of Pathology, Chifeng Municipal Hospital, Inner Mongolia, China; 3 Geneis Beijing Co., Ltd., Beijing, China; 4 Academician Workstation, Changsha Medical University, Changsha, China

**Keywords:** deep learning, gastrointestinal cancers, molecular biomarker, multimodal data, pathological image

## Abstract

**Introduction:**

Deep learning (DL) shows great potential for predicting biomarkers from routine histopathological slides of gastrointestinal (GI) cancers. Yet most existing models are validated on limited patient cohorts, while pathological image annotation and molecular marker standardization demand substantial professional expertise. To address these gaps, we constructed the Gastrointestinal Cancer Pathological Image Archive (GICPIdb, gicpidb.shubuzuo.top), a dedicated database and web platform covering seven major GI cancer types.

**Methods:**

High-quality hematoxylin and eosin (H&E)-stained whole-slide images were collected from multiple sources and uniformly processed. Image annotations were performed by board-certified pathologists following standardized protocols. GICPIdb offers five interactive web modules for data uploading, quality control, feature extraction, online annotation and AI-based prediction. Its intuitive interface supports data browsing, retrieval, visualization and downloading.

**Results:**

The database houses 2,863 pathologist-annotated, uniformly processed, high-quality H&E stained images collected from 2,655 patients. Of these, 1,699 patients were sourced from The Cancer Genome Atlas (TCGA), 182 from the Clinical Proteomic Tumor Analysis Consortium (CPTAC), and 424 from China-Japan Friendship Hospital and 350 from Chifeng Municipal Hospital in Inner Mongolia, China. It also integrates data on over 50 key molecular markers (e.g., MSI, TMB) and prognostic labels related to survival, recurrence and metastasis.

**Discussion:**

GICPIdb aims to promote the development of DL-driven AI tools for cancer research and clinical translation. The multi-institutional data collection and standardized annotation pipeline are expected to enhance the generalizability and reproducibility of AI-based prediction models across diverse patient populations.

## Introduction

Gastrointestinal (GI) cancers, including esophageal, gastric, colorectal, liver, and pancreatic cancers, are among the most prevalent malignant tumors. In 2026, GI cancers accounted for 17.49% of all new invasive cancer cases and 28.54% of cancer-related deaths in the United States (Siegel et al., 2026). According to the 2022 China Cancer Statistics Report, there has been a gradual decline in the incidence rates of stomach, liver, and esophageal cancers. Conversely, a significant increase in the incidence of colorectal cancer has been observed across the population (Xia et al., 2022).

The digitization of histopathology slides via whole-slide imaging (WSI) has catalyzed the rapid development of computational pathology, wherein deep learning methods are increasingly applied to tasks ranging from cellular-level segmentation and detection to whole-slide diagnosis, molecular profiling, and prognostic prediction. To reduce the reliance on labor-intensive pixel-level annotations, Guo et al. (2023) proposed SAC-Net, a weakly supervised nuclei segmentation framework requiring only centroid annotations that employs a constraint–attention triple-network architecture with Confident Learning refinement to achieve performance competitive with fully supervised methods. Wang et al. (2023a) developed FMDet, a generalizable mitosis detection algorithm that reformulates object detection as semantic segmentation with Fourier-based domain augmentation, ranking first in the MIDOG 2021 challenge and demonstrating robust cross-center generalization. Beyond task-specific models, Wang et al. (2024) introduced CHIEF, a general-purpose pathology foundation model combining unsupervised and weakly supervised pretraining on 60,530 WSIs across 19 anatomical sites, which outperformed state-of-the-art methods by up to 36.1% across 24 international validation cohorts in cancer detection, tumor origin identification, and survival prediction. Building upon foundation model representations, Yuan et al. (2025) proposed PROGPath, a unified multimodal framework that integrates histopathological image features with clinical variables via attention-guided multiple-instance learning and a cross-attention transformer. PROGPath achieved consistently superior pan-cancer prognosis prediction across 17 external cohorts. Additionally, Cai et al. (2024) released HiCervix, the largest multi-center cervical cytology dataset comprising 40,229 cells across 29 hierarchical classes, with the HierSwin benchmark achieving 82.93% averaged accuracy and outperforming board-certified cytopathologists. Collectively, these advance, spanning weakly supervised segmentation, domain-generalizable detection, foundation models, multimodal prognostication, and large-scale benchmarking, have established a robust methodological and data foundation for AI-driven histopathology image analysis.

Despite these advances, several challenges continue to impede the clinical translation of DL-based pathology models, particularly for GI cancers. A primary obstacle is the scarcity of publicly available, high-quality pathology images linked to diagnostic, prognostic, and molecular sequencing data (Cui and Zhang, 2021; Spanhol et al., 2015; He et al., 2025; Ye et al., 2022). The manual annotation of WSIs is a labor-intensive and costly process that complicates data acquisition (Bera et al., 2019; Madabhushi and Lee, 2016; Baxi et al., 2022; Mao et al., 2025). Additionally, the lack of digital pathology data from diverse cohorts restricts the development of validated DL models that can be applied broadly across different population groups (Marra et al., 2025). Most publicly available digital pathology datasets originate from the United States and European countries, resulting in a notable absence of East Asian population samples. Furthermore, the lack of standard criteria for image quality control, online annotation, and an integrative platform for comparing various DL-based prediction methods presents additional challenges. These issues underscore the urgent need for standardization efforts to enhance the accuracy and reproducibility of DL-based cancer research.

In response to these challenges, we presented GICPIdb (Gastrointestinal Cancers Pathological Image Archive), a rigorously curated multimodal database tailored for GI cancers pathology images. GICPIdb assembled raw pathological images, clinical data, and gene sequencing information across seven GI cancers, employing a standardized analysis workflow to develop a comprehensive repository of meticulously annotated, high-quality pathological image data. Additionally, it included information on over 50 molecular markers for each patient. The platform featured a user-friendly interface that facilitated browsing, searching, visualizing, and downloading pathological data. Furthermore, GICPIdb provided five interactive image analysis tools to assist users in managing and analyzing their own histological imaging data. This database was positioned as an invaluable resource for researchers and clinicians in the field of digital pathology.

## Materials and methods

### Data collection

Formalin-Fixed Paraffin-Embedded (FFPE) slides are regarded as the gold standard in diagnostic medicine due to their high image quality, which facilitates accurate computational analysis. In this study, we systematically collected raw FFPE slides from four sources: The Cancer Genome Atlas (TCGA), the Clinical Proteomic Tumor Analysis Consortium (CPTAC), The China-Japan Friendship, and Chifeng Municipal Hospital in Inner Mongolia, China. For the TCGA cohorts, raw FFPE slides, clinical data, and gene sequencing information were downloaded from GDC Portal (https://portal.gdc.cancer.gov/). In the CPTAC cohorts, FFPE slides were obtained from The Cancer Imaging Archive (https://www.cancerimagingarchive.net/), while clinical and gene sequencing data were retrieved from the same GDC Portal (https://portal.gdc.cancer.gov/). The China-Japan Friendship and Chifeng cohort consisted of patients who underwent primary GI cancers resection surgery at China-Japan Friendship and Chifeng Municipal Hospital between 2020 and 2025. This study received approval from the China-Japan Friendship Hospital Committee (Approval Number: 2025-KY-357 and 2024-KY-329-1) and Chifeng Municipal Hospital Committee (Approval Number: CK2023054). Pathological slides from TCGA cohort were scanned using the Aperio ScanScope CS system at a resolution of 0.25 μm/pixel (Cruz-Roa et al., 2017; Zormpas-Petridis et al., 2021; Azarianpour et al., 2025). For CPTAC public dataset, the scanner model was not explicitly documented; however, the scanning resolution was recorded as 0.2527 μm/pixel (Azarianpour et al., 2025). For surgically resected tumors from the Chifeng and China-Japan Friendship Hospital cohorts, representative hematoxylin and eosin (H&E)-stained slides were scanned at 40 × magnification using a PRECICE 500 scanner (NuVasive, United States) at a fixed resolution of 0.25 μm/pixel. Across all cohorts, slides were scanned at resolutions of at least 0.25 μm/pixel (Hanna et al., 2022), ensuring comparable image quality for downstream analysis.

### Pathology image data processing

A standardized pathology image analysis workflow was employed to process the raw FFPE slides, encompassing WSI filtering, annotation of tumor tissue regions, quality control, image patching and filtering, color normalization and feature extraction.

- **WSI filtering and annotation of tumor tissue regions**:

(1) Standardization of the annotation pipeline: All pathological images were first reviewed in full by three senior pathologists, each with over 10 years of experience in gastrointestinal pathology, to exclude slides lacking tumor tissue or representing non-target tumor types (e.g., non-invasive intraductal papillary mucinous neoplasm, acinar cell carcinoma, and neuroendocrine tumor). Tumor ROIs were manually delineated using ASAP software, and annotation vertex coordinates (X, Y) were exported in XML format. Necrosis, blood clots, tissue overlap and folding, carcinoma *in situ*, and benign stromal and epithelial regions were explicitly excluded to prevent contamination of training data by non-tumor elements.

(2) Formal assessment of interobserver agreement: One hundred slides were randomly selected, and each was independently annotated by two pathologists. Interobserver agreement was quantified using the Dice similarity coefficient (DSC) (Jager et al., 2023). The complete DSC formula: DSC = 2 × |A ∩ B| / (|A| + |B|), where |A ∩ B| denotes the area of overlap between the two annotators. The DSC ranges from 0 to 1, with higher values indicating greater spatial overlap between two annotations and, correspondingly, better interobserver agreement: 0 denotes no overlap, and 1 denotes perfect overlap. The mean DSC was 0.87, with a range of 0.78 - 0.94.

(3) Quality control and review mechanism: Beyond the formal interobserver agreement assessment, all remaining slides were annotated under a dual-signoff protocol: one pathologist performed the initial annotation and a second independently reviewed it. When the reviewing pathologist raised concerns regarding boundary delineation or grading, a three-person panel was convened to reach a consensus. This mechanism ensures annotation quality across all slides.

(4) Annotation precision specifications. Following the protocol of Jager et al. (2023) annotation precision was standardized to a spatial resolution on the order of 1–2 mm, with a contour line width of 0.2 mm, thereby avoiding the excessive time cost and spurious variability introduced by overly fine-grained delineation.

- **Quality control**: Digital pathology image analysis requires high-quality input images. To ensure image quality, we automatically assess histology slides using HistoQC (Janowczyk et al., 2019) software to obtain quality control metrics.
- **Image patching and filtering**: To mitigate the impact of background noise and invalid regions on pathological image analysis and feature extraction. All slides were decomposed into non-overlapping 256 × 256 pixel patches at a resolution of 0.5 μm/pixel. A tissue coverage threshold of 70% was applied to discard patches with insufficient tissue content, blank regions, or imaging artifacts.
- **Color normalization**: Multicenter pathological images exhibit substantial color heterogeneity due to variations in scanning devices and staining batches, which may induce domain shifts and reduce cross-cohort comparability. To address this, color normalization was applied to all qualified image patches using the Macenko (Macenko et al., 2009) algorithm, as implemented in the open-source TIAToolbox (Pocock et al., 2022). This procedure standardized staining intensity and color distribution, thereby reducing systematic biases introduced by different scanners and staining batches and yielding visually consistent pathological images across cohorts for subsequent analysis
- **Features extraction:** Multiple feature extraction approaches were employed for patch-level representation learning, encompassing both deep learning-based and traditional handcrafted features.

(1) CLAM (Lu et al., 2021): An ImageNet-pretrained ResNet-50 backbone, truncated after the third residual stage and followed by an adaptive average pooling layer, was used to extract 1,024-dimensional feature vectors from each tissue patch (256 × 256 pixels).

(2) RetCCL (Wang et al., 2023b): RetCCL is a contrastive self-supervised learning model based on the MoCo v3 framework with clustering-guided contrastive loss, pretrained on approximately 15 million histopathology image tiles from 32,000 whole-slide images. The model employs a ResNet-50 backbone and was used to extract 2,048-dimensional feature embeddings per tile via the final global average pooling (GAP) layer.

(3) CTransPath (Wang et al., 2022): CTransPath is a Swin Transformer-based encoder pretrained via MoCo v3-driven semantic contrastive learning on approximately 15 million pathology tiles. With pretrained weights frozen, the encoder was used to extract 768-dimensional tile-level embeddings.

(4) Traditional handcrafted features: For comparison with deep learning-based representations, classical color and texture features were also extracted. For texture characterization, the wavelet multi-sub-band co-occurrence matrix (WMCM) method was employed, in which multilevel wavelet decomposition was first applied to obtain sub-bands (LL, LH, HL, and HH), followed by the computation of gray-level co-occurrence matrices (GLCMs) for each sub-band. Statistical descriptors were then derived as texture features. For color characterization, low-order color moments were computed for each of the R, G, and B channels: the first-order moment (mean), second-order moment (standard deviation), and third-order moment (skewness), yielding a 9-dimensional color feature vector.

### Definition of prognostic events

Overall survival (OS) was defined as the time from surgery to death from any cause. Progression-free interval (PFI) refers to the time until the first occurrence of a new tumor event, which includes disease progression, locoregional recurrence, distant metastasis, new primary tumors, or death with tumor presence. Disease-free survival (DFS) is the interval from surgery to tumor recurrence, encompassing local-regional, peritoneal, and distant recurrences, as well as death, or until the date when the patient was last known to be free of progression. Disease-Specific Survival (DSS) is defined as the time from the initial diagnosis or surgery to death specifically attributable to the disease. The duration to recurrence or metastasis was calculated from the time of surgery to the point at which recurrence or metastasis was diagnosed during follow-up.

### Identification of molecular markers

- Tumor Mutational Burden (TMB): TMB was defined as the total number of non-synonymous mutations detected across the whole-exome sequence. Counts of non-synonymous mutations (Missense_Mutation, Nonsense_Mutation, Frame_Shift_Del, Frame_Shift_Ins, In_Frame_Del, In_Frame_Ins, Nonstop_Mutation, Splice_Site, Translation_Start_Site) were computed from MAF files using the R package maftools (Mayakonda et al., 2018). The high TMB threshold was set to the 25th percentile within each cancer-type cohort; this percentile cutoff was adopted following previous studies (Wang et al., 2021; Zhou et al., 2019; Kowanetz et al., 2017). Samples lacking available MAF files were assigned not available (NA).
- Microsatellite Instability (MSI): MSI is a core molecular biomarker in cancer genomics, widely used to identify impaired DNA mismatch repair fidelity in tumor cells. MSI status annotations for the TCGA cohort were extracted from a prior published study (doi: 10.1038/s41591-019-0462-y) MSI datasets for the CPTAC cohort were downloaded from the cBioPortal database (https://www.cbioportal.org/). For clinical specimens collected at China-Japan Friendship Hospital and Chifeng Municipal Hospital, MSI classification was assessed via immunohistochemistry (IHC) targeting mismatch repair (MMR) proteins. The grouping criteria are specified below: samples with intact, concurrent expression of both MSH2 and PMS2, or simultaneous positive staining for MLH1 and MSH6, were classified as MSI-high (MSI-H). All other MMR protein expression profiles were defined as microsatellite stable (MSS). Specimens with incomplete or unevaluable MMR/MSI results were labeled NA.
- Homologous recombination deficiency (HRD): HRD scores were obtained from a previous study (Knijnenburg et al., 2018). An HRD positivity threshold of 42 was adopted, consistent with prior studies (Wang Y. et al., 2025; Sharma et al., 2018).

Furthermore, Supplementary Table S1 provides a complete list of more than 50 markers, together with detailed descriptions of their sources, definitions, and missing-value handling.

### AI Predictor module performance evaluation

GICPIdb incorporates an AI Predictor module powered by deep learning models pretrained on TCGA-CRC and TCGA-STAD cohorts in our previous studies (Chang et al., 2026; Wang W. et al., 2025), enabling direct prediction of MSI status and TMB from histology images. Model performance was evaluated using the area under the receiver operating characteristic curve (AUC), accuracy, sensitivity, specificity, precision, F1-score, and calibration. Sensitivity, specificity, accuracy, precision, and F1-score were derived from the confusion matrix:


$$\begin{array}{l} Sensitivity=\frac{TP}{TP+\;FN} \end{array}$$

$$\begin{array}{l} Specificity=\frac{TN}{TN+FP} \end{array}$$

$$\begin{array}{l} Accuracy=\frac{TP+TN}{TP+TN+FP+FN} \end{array}$$

$$\begin{array}{l} Precision=\frac{TP}{TP+FP} \end{array}$$

$$\begin{array}{l} F1-score=2\frac{precision\;x\;recall\;}{precision\;+\;recall} \end{array}$$

Where, TP, TN, FP, and FN denote true positives, true negatives, false positives, and false negatives, respectively. AUC was computed from the ROC curve. Model calibration was assessed using the Brier score and the expected calibration error (ECE) with 10 equal-width bins, calculated via scikit-learn.

### Web portal

The front-end display of the GICPIdb was implemented using HTML and CSS, while the back-end data management was handled by the PostgreSQL database (version 15.3) with management system version 8.0.20. Interaction between the front-end and back-end was facilitated through JavaScript and C#. All charts within GICPIdb were generated using ECharts and Plotly.js. The GICPIdb database was deployed on a Nginx server and was freely accessible. Comprehensive testing of all GICPIdb functions was conducted on Google Chrome, Firefox, Microsoft Edge, and Apple Safari browsers.

## Results

### Overview of GICPIdb

**Figure 1:**
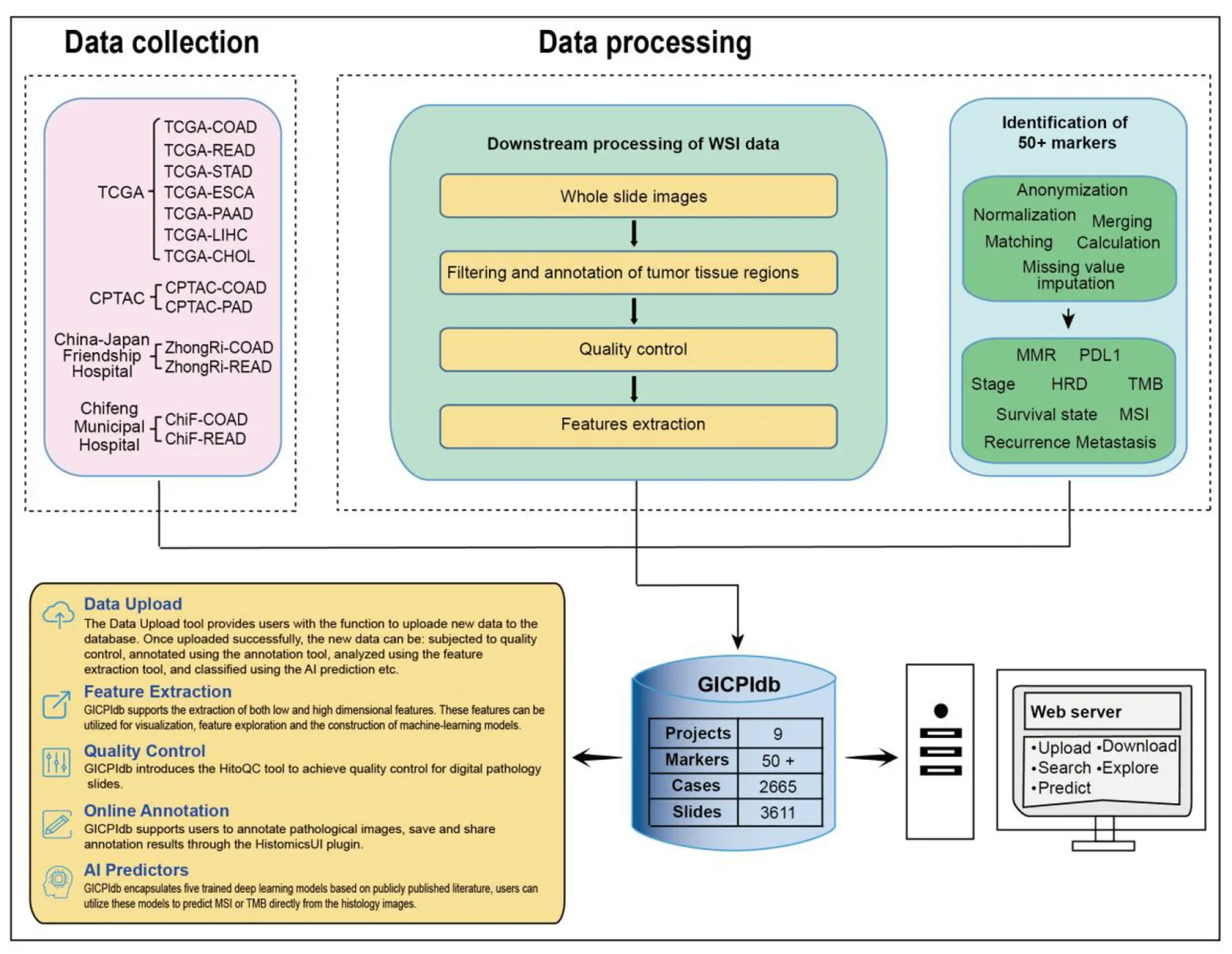
Schematic representation of the GICPIdb portal.

We collected raw pathological images, clinical data, and gene sequencing data for GI cancers from multiple sources, including TCGA, CPTAC, China-Japan Friendship Hospital and Chifeng Municipal Hospital in Inner Mongolia, China. The overall workflow of GICPIdb is illustrated in Figure 1. Initially, slides were manually reviewed by three pathologists, who removed those without tumor tissue or that did not represent GI cancers. The remaining slides underwent manual annotation of tumor regions by the pathologists, followed by quality control and feature extraction from the images. All result files are freely accessible to users. The latest release of GICPIdb includes 2,863 well-annotated high-quality FFPE slides from 2,655 patients, encompassing seven major types of GI cancers. For each patient, over 50 molecular markers and prognostic classification labels have been identified. The distribution of cases and slides stratified by key molecular and clinicopathological features is presented in Supplementary Table S2 and Figure S1.

### Browsing module

**Figure 2:**
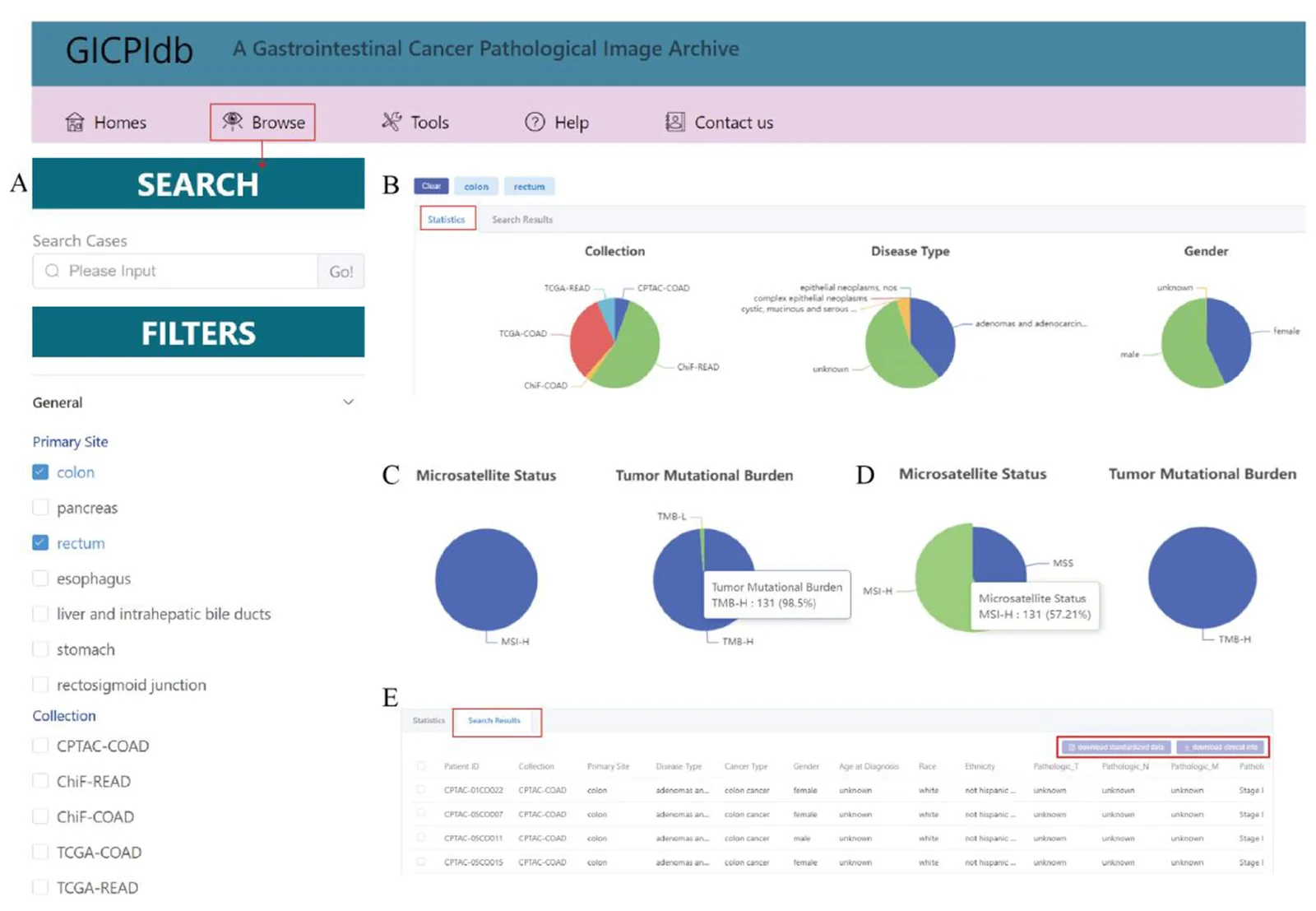
User interface for data browsing. **(A)** Query search criteria. **(B)** Summary statistics of the data content based on the query. **(C)** Distribution of Tumor Mutational Burden-High (TMB-H) and Tumor Mutational Burden-Low (TMB-L) among Microsatellite Instability-High (MSI-H) patients. **(D)** Distribution of MSI-H and Microsatellite Stable (MSS) in TMB-H patients. **(E)** Display of query results.

We developed a user-friendly browsing module to enable quick data retrieval from the GICPIdb database. Users can query the database by inputting one or more case IDs. GICPIdb offers basic filters, including general, clinical, pathological, molecular, and prognostic information, to refine query results and help users quickly identify samples of interest (Figure 2A). The “Statistics” tab visualizes the query results (Figure 2B).

In addition to facilitating rapid data retrieval, the module allows users to conduct straightforward data exploration. For instance, our analysis reveals a significant correlation between patients with high microsatellite instability (MSI-H) and those with high tumor mutation burden (TMB-H), with 98.5% of MSI-H patients exhibiting TMB-H characteristics (Figure 2C). Conversely, within the TMB-H patient cohort, MSI-H and microsatellite stable (MSS) patients appear to have a relatively equitable distribution (Figure 2D).

The “Search Results” table provides comprehensive clinicopathological details for each sample, including primary site, disease type, gender, pathologic stage, and other relevant information (Figure 2E). In addition to browsing, users can perform additional operations, such as downloading data and visualizing images.

For search results, users can download data for one or more samples by selecting the “Download Standardized Data” or “Download Clinical Info” buttons. GICPIdb provides several data categories for download, including tumor region annotations, CLAM feature files, HistoQC quality control files, color and texture feature files, and clinical data. These categories provide valuable insights into the molecular and pathological characteristics of each sample, establishing GICPIdb as a comprehensive resource for cancer research and clinical applications. For example, tumor area annotation files facilitate the development of automated tools for accurate and efficient WSI annotation. In addition, *t*-SNE visualization was performed to compare the sample-level feature distributions extracted by different feature encoders, demonstrating that RetCCL exhibited superior discriminative capability in stratifying samples by MSI and TMB status (Figure S2). Furthermore, by leveraging the precomputed features, researchers can directly develop new models and algorithms without downloading and processing large raw histology image datasets.

### Application modules

**Figure 3:**
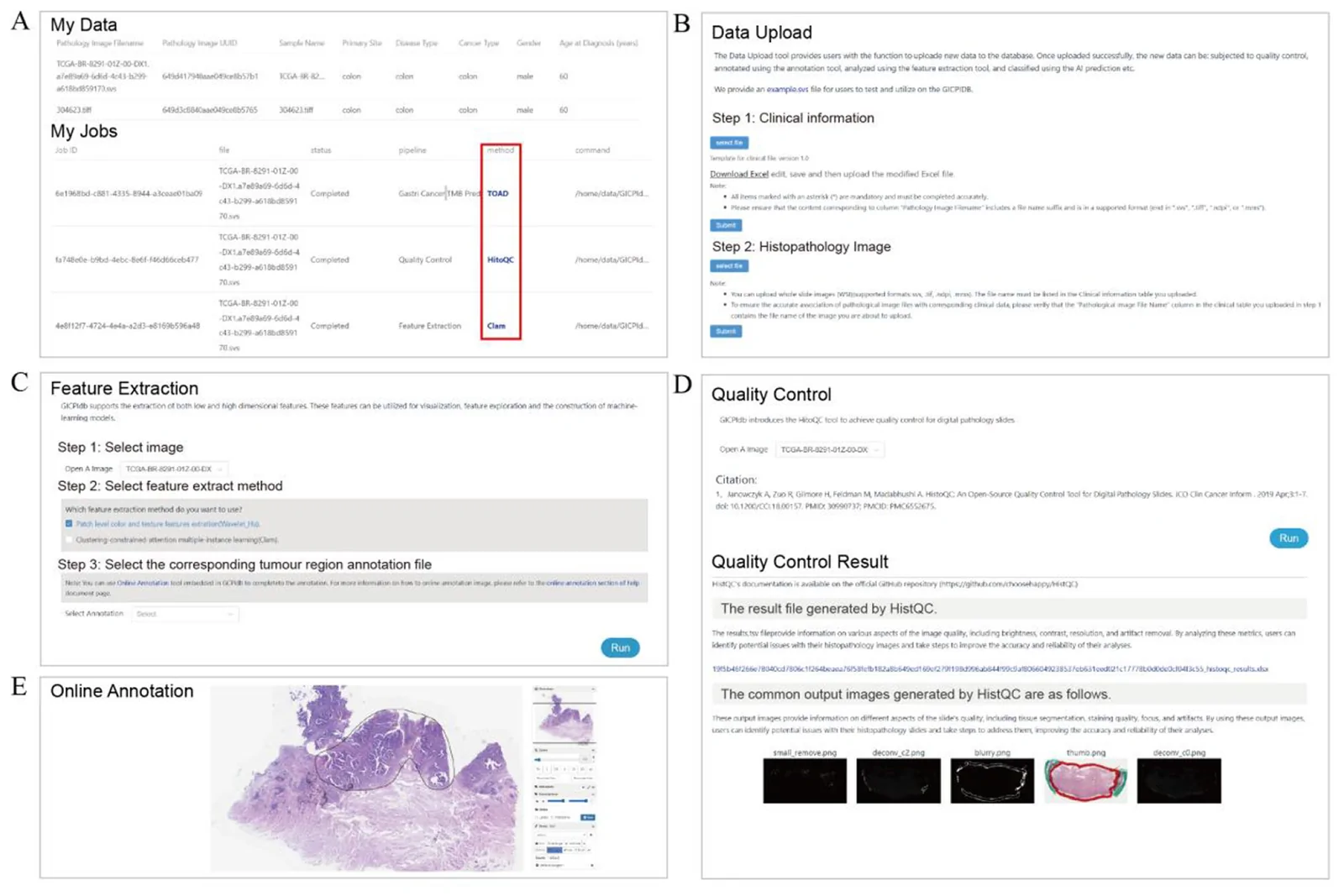
Application modules of GICPIdb. **(A)** Historical data and task detail information submitted by users. **(B)** Data upload interface. **(C)** Feature extraction interface. **(D)** Quality control interface. **(E)** Online annotation interface.

GICPIdb offers five web-based interactive analysis tools to facilitate downstream analysis. To ensure the security of users' private data, GICPIdb has implemented user registration and login functionality. Users can conveniently register or access their personal information by clicking on the login or username option in the upper right corner of the interface. The “My Data” menu provides a comprehensive list of all data uploaded by the user, including clinical information and the universally unique identifier (UUID) assigned by GICPIdb to each pathological image. The “My Jobs” menu displays a detailed list of the user's historical task information, including the job UUID, runtime, status, pipeline, method, and run logs. Users can access detailed result information by clicking on the corresponding method for each task (Figure 3A).

Data Upload tool: This tool allows users to upload new data to GICPIdb (Figure 3B). Successfully uploaded data can be utilized for downstream analyses.

Feature Extraction tool: GICPIdb provides two methods for feature extraction. These features can be used for visualization, feature exploration, and the development of AI models (Figure 3C). Feature vectors extracted from image blocks and the corresponding coordinates of the upper left corner of each block in the original image are typically stored in H5 files.

Quality Control tool: GICPIdb introduces the HistoQC tool, which automates quality control for digital pathology slides (Figure 3D). The result files and output images generated by HistoQC assess various aspects of image quality, including brightness, contrast, resolution, artifact removal, and staining quality. By analyzing these metrics, users can identify potential issues with their histopathology images and take steps to enhance the accuracy and reliability of their analyses.

Online Annotation tool: GICPIdb enables users to annotate, save, and share annotations online using the HistomicsUI (Gutman et al., 2017) plugin (Figure 3E).

AI Predictor tool: GICPIdb incorporates trained deep learning models based on our previous studies, which can be utilized to predict MSI, TMB directly from histology images. The CRC MSI prediction model achieved an AUC of 0.858 on the TCGA-CRC internal test set and AUCs of 0.784, 0.803, and 0.753 on CPTAC-CRC, ChiF-CRC, and ZhongRi-CRC external cohorts, respectively. The CRC TMB prediction model yielded an AUC of 0.856 on TCGA-CRC. For GC MSI prediction, the model achieved an AUC of 0.828 on TCGA-STAD. Two GC TMB prediction models were evaluated on TCGA-STAD, attaining AUCs of 0.836 Chang et al. (2026) and 0.767 (TOAD). All models exhibited consistent discriminative performance across cohorts, with detailed metrics summarized in Supplementary Table S3.

**Figure 4:**
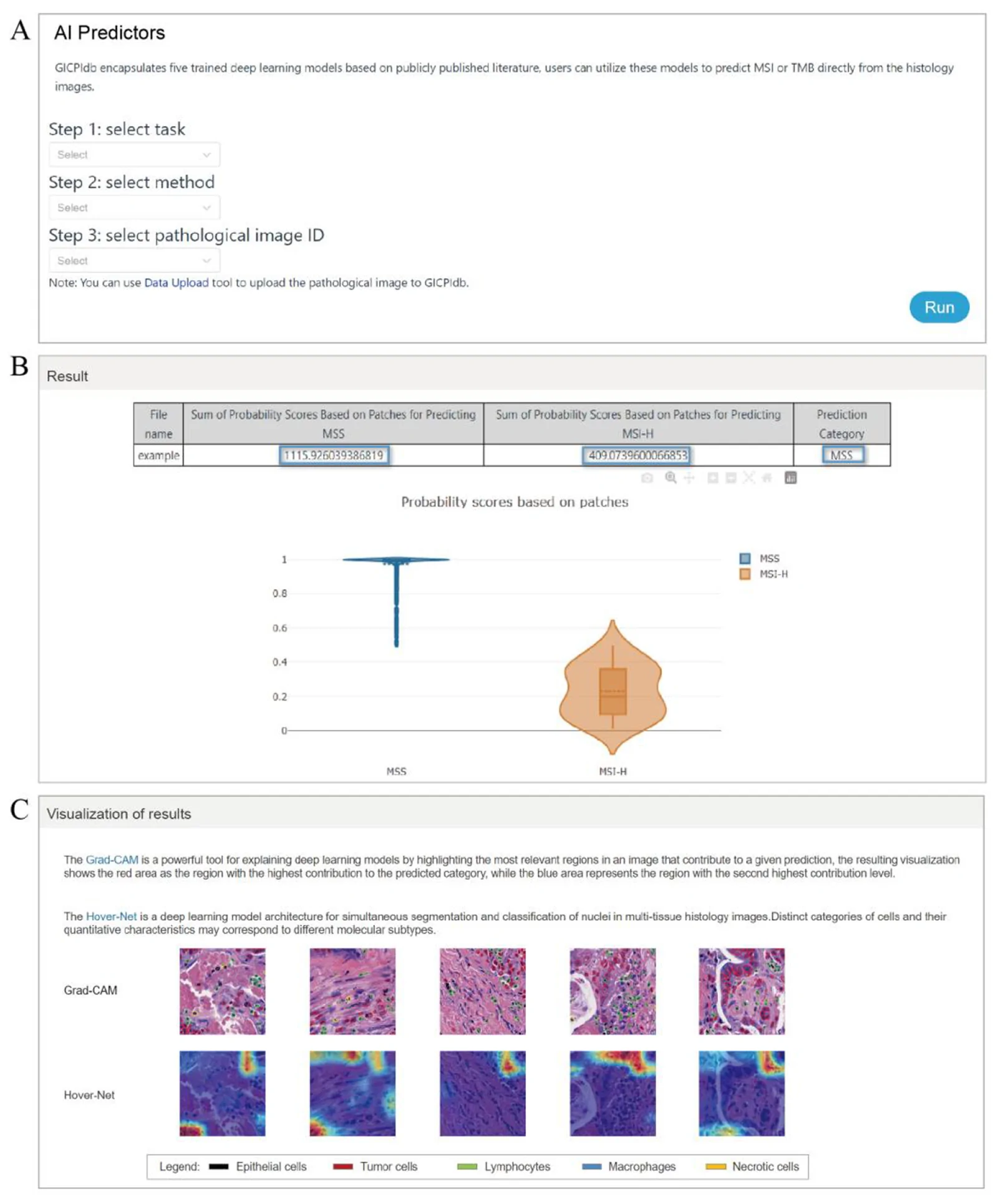
Interface for AI predictors. **(A)** AI predictor page. **(B)** Violin plot displaying scores for each category. **(C)** Grad-CAM visualization results alongside Hover-Net cell segmentation outcomes.

Before initiating the task, the AI Predictor tool requires users to select the pathological image and the corresponding tumor region annotation file (Figure 4A). When users opt for the “NM Model” during Step 2, the AI Predictor provides scores for each category (Figure 4B), along with Grad-CAM (Wall et al., 2026) results and Hover-Net (Graham et al., 2019) cell segmentation results (Figure 4C). Grad-CAM highlights regions with high relevance to the predictions, aiding in the interpretation of the deep learning models. Hover-Net is a deep learning architecture designed for simultaneous segmentation and classification of nuclei in multi-tissue histology images. Notably, our findings indicate that the model's visualizations suggest it focuses on lymphocytes, stroma, and cancer regions when predicting MSI-H.

If users select the “TOAD” option during Step 2, the AI Predictor furnishes the final predictive category result. With its capacity to predict MSI or TMB directly from histology images, the AI Predictor serves as a valuable tool for cancer research and clinical applications, providing users with a powerful resource to enhance their research and inform clinical decision-making processes.

### Comparison of GICPIdb with existing publicly available pathology databases

We systematically compared GICPIdb with seven publicly available pathology databases (TCGA, TCIA/CPTAC, CAMELYON16/17, PANDA, LC25000, PanNuke, and HISTAI) across multiple dimensions, including primary focus, image format, dataset scale, annotation type, molecular marker availability, standardized processing, precomputed features, and online analysis capability (Supplementary Table S4). GICPIdb is a comprehensive publicly accessible pathology database dedicated to gastrointestinal cancer, covering seven major GI cancer types with 2,863 WSIs from 2,655 patients across four data sources. Unlike existing pan-cancer archives (TCGA, TCIA/CPTAC, and HISTAI) that distribute raw slides without integrated molecular or prognostic curation, and single-organ datasets (CAMELYON16/17, PANDA, and LC25000) that lack molecular marker annotations entirely, GICPIdb uniquely integrates over 50 molecular markers (MSI, TMB, HRD, etc.) and six prognostic endpoints (OS, PFI, DFS, DSS, recurrence, metastasis) with manually annotated tumor regions of interest. Furthermore, its standardized preprocessing pipeline, comprising HistoQC quality control, Macenko color normalization, and systematic patching, along with precomputed features from three deep learning extractors (CLAM, RetCCL, and CTransPath) and an online analysis platform with built-in MSI/TMB prediction models, provides a valuable resource for lowering the computational barrier to pathology-AI development and accelerating the clinical translation of computational pathology in GI oncology.

### Summary and future developments

Advances in digital pathology and AI have provided new insights and demonstrated significant promise for tumor diagnosis, prognosis, and therapeutic response prediction (Bera et al., 2019). GICPIdb is a comprehensive publicly accessible pathological image database dedicated to gastrointestinal cancer, offering a vast collection of standardized, manually annotated, high-quality pathology data encompassing over 50 markers. With respect to TMB, the database retains the raw non-synonymous mutation counts for all enrolled samples, enabling researchers to define high-TMB subgroups using either established pan-cancer thresholds [e.g., 10 mut/Mb (Bao et al., 2020; Zhang et al., 2021) or 20 mut/Mb (Chalmers et al., 2017)] or cancer-type-specific quantile cutoffs reported in the literature (Budczies et al., 2019). MSI status was determined through a multi-platform parallel assessment strategy: MSI classification for TCGA and CPTAC cohort samples was performed using next-generation sequencing (NGS) data, whereas clinical specimens from China-Japan Friendship Hospital and Chifeng Municipal People's Hospital were evaluated via IHC detection of MMR protein expression. Given the multi-center composition of this database, inherent heterogeneity across the detection platforms employed by different institutions introduces unavoidable cross-cohort bias, a limitation intrinsic to the construction of databases integrating real-world, multi-source pathological data. In addition, we have developed a user-friendly interface that facilitates browsing, searching, visualizing, analyzing, and downloading data. Furthermore, GICPIdb provides a suite of user-friendly and flexible image analysis tools for storing, managing, and analyzing users' own histology imaging data.

*t*-SNE visualization revealed that none of the evaluated feature encoders could clearly separate samples by MSI or TMB status. Several factors may account for this observation. First, *t*-SNE primarily preserves local neighborhood structure rather than global class boundaries; thus, visual overlap in the embedding space does not necessarily imply a lack of discriminative information in the features. Second, MSI and TMB are molecular phenotypes that may not exhibit pronounced, spatially uniform histomorphological patterns discernible at the WSI level. Third, aggregating patch-level features into slide-level representations may dilute sparse, focal morphological signals associated with these molecular traits. Moreover, inter-patient histological heterogeneity and the complexity of the tumor microenvironment can further obscure weak MSI/TMB-correlated signals. In future work, we plan to investigate attention-guided patch-level dissection and supervised contrastive learning to derive feature representations with improved discriminative capability for molecular stratification.

In the future, we plan to periodically update GICPIdb in several key areas: First, we aim to expand the datasets by adding more standardized, high-quality pathological image data. Second, we will continue to identify additional marker tags, including molecular subtypes (Gao et al., 2019; Zheng et al., 2022), radiotherapy (Lou et al., 2022), immunotherapy (Bilal et al., 2023), and neoadjuvant (Joo et al., 2021) response to enhance the data's reuse value. Third, we recognize that our current annotations are relatively coarse; thus, we plan to improve annotation accuracy to the cellular or nuclear level, incorporating nuclear and cell type annotations. Fourth, although the embedded AI prediction module enables prediction of tumor TMB and MSI status, its performance requires further improvement. In particular, the predictive models for gastric cancer MSI and TMB need to be validated using larger, independent cohorts. Finally, we will update image processing pipelines and provide advanced analysis algorithms, including lymphocyte detection, tumor-infiltrating lymphocyte (TIL) quantification (Swiderska-Chadaj et al., 2019; Saltz et al., 2018; Amgad et al., 2020) and nuclear segmentation (Chen et al., 2022).

Domain shift arising from inter-center heterogeneity in staining protocols and scanner parameters is a well-recognized challenge in multi-institutional studies, undermining cross-cohort comparability. While the Macenko algorithm was applied to normalize staining intensity and color distribution across all WSIs, computational correction alone cannot fully eliminate intrinsic staining variability. To address this prospectively, we will collaborate with partner hospitals to establish unified staining specifications across participating centers, with a comprehensive standard operating procedure (SOP) to be developed in future work.

We will establish a dedicated multidisciplinary data management team responsible for data acquisition, quality control, and end-user technical support. Financial sustainability will be ensured through institutional funding, research grants, and collaborative resources. GICPIdb will undergo major updates annually, incorporating newly curated datasets, updated annotation files, and refined analysis workflows. All releases will follow semantic versioning (e.g., v1.0.0), and each version will be permanently archived to ensure full traceability and reproducibility. All GICPIdb data were collected under IRB-approved protocols. Informed consent was obtained from all participants, and personally identifiable information has been rigorously de-identified prior to inclusion.

In summary, GICPIdb is a comprehensive data repository that houses high-quality and meticulously annotated pathological data. It serves as a valuable tool by offering a user-friendly platform for efficient data analysis and visualization. We are confident that with ongoing updates, GICPIdb will play a significant role in speeding up the development of deep learning-based predictive models and facilitating the practical application of digital pathology data in GI cancers research.

## Data Availability

The original contributions presented in the study are included in the article/Supplementary material, further inquiries can be directed to the corresponding authors.
