# Supplementary material for: GICPIdb: an archival repository of multimodal data focusing on pathological images for gastrointestinal cancers
Source: Front Big Data. 2026 Aug 31;9:1924721. doi: 10.3389/fdata.2026.1924721 (PMC13572243; doi:10.3389/fdata.2026.1924721)
Supplement: Supplementary file 1 [file Supplementary_file_1.docx]

**Figure S1. Distribution of cases and slides stratified by key molecular and clinicopathological features.** (A) Cases distribution. (B) Slides distribution.

**Figure S2. t-SNE visualization of sample-level feature distributions from different feature encoders, stratified by MSI and TMB status.**
